# Symptom profile in partial responders to a proton pump inhibitor compared with treatment-naïve patients with gastroesophageal reflux disease: a *post hoc* analysis of two study populations

**DOI:** 10.1186/1471-230X-14-177

**Published:** 2014-10-10

**Authors:** Nimish Vakil, Anna Niklasson, Hans Denison, Anna Rydén

**Affiliations:** University of Wisconsin School of Medicine and Public Health, Aurora Summit Hospital, 36500 Aurora Drive, Summit, WI 53066 USA; AstraZeneca R&D, Mölndal, Sweden

**Keywords:** Abdominal pain, Constipation, Heartburn, Proton pump inhibitors, Regurgitation, Gastric

## Abstract

**Background:**

Partial response to proton pump inhibitor (PPI) therapy poses a healthcare challenge. This study aimed to compare symptom profiles in partial PPI responders and treatment-naïve patients with gastroesophageal reflux disease (GERD).

**Methods:**

A *post hoc* analysis of data from two studies was performed. Partial PPI responders with GERD (n = 580; NCT00703534) had frequent (≥ 3 days/week) heartburn and/or regurgitation despite PPI therapy; patients with no improvement were excluded. Treatment-naïve patients with GERD (diagnosed by endoscopy and pH-metry; n = 203; NCT00291746) had frequent (≥ 3 days/week) upper gastrointestinal symptoms. The Gastrointestinal Symptom Rating Scale (GSRS) was completed by all patients at study entry and by treatment-naïve patients after PPI therapy.

**Results:**

The highest (mean [95% confidence interval]) discomfort scores were reported in the Reflux (heartburn, regurgitation), Indigestion, and Abdominal pain domains of the GSRS, both in partial PPI responders (4.3 [4.2–4.4], 3.7 [3.6–3.8], and 3.4 [3.3–3.5], respectively) and in treatment-naïve patients (3.5 [3.3–3.7], 3.6 [3.4–3.7], and 3.1 [3.0–3.3], respectively). Partial PPI responders reported more discomfort than treatment-naïve patients in the Reflux, Abdominal pain, and Constipation domains (4.3 [4.2–4.4] vs. 3.5 [3.3–3.7], 3.4 [3.3–3.5] vs. 3.1 [3.0–3.3], and 2.5 [2.4–2.6] vs. 2.1 [1.9–2.2], respectively). All GSRS domain scores improved in treatment-naïve patients following PPI therapy.

**Conclusions:**

Symptom patterns in partial PPI responders were similar to those in treatment-naïve patients with GERD, but partial PPI responders experienced more severe reflux, abdominal pain, and constipation than did treatment-naïve patients.

## Background

About 20% to 30% of patients with gastroesophageal reflux disease (GERD) experience persistent, troublesome heartburn or regurgitation despite proton pump inhibitor (PPI) therapy [1]. These patients pose a challenge to the healthcare system and the treating physician, and their management is expensive [2]. There are several possible explanations for residual reflux symptoms on PPI therapy [3]. Persistent reflux of acidic and weakly acidic content has been described in patients with residual reflux symptoms [3, 4]. Delayed gastric emptying may predispose to persistent reflux in some patients with GERD [3]. Some studies have suggested that symptoms may also be caused by non-GERD conditions (e.g. dyspepsia and irritable bowel syndrome [IBS]) [5]. Esophageal visceral hypersensitivity may result in symptom reporting under physiologic conditions [3]. Poor adherence to treatment recommendations, such as taking the PPI at an inappropriate time or not adhering properly to a regular PPI schedule, may also explain persistent symptoms in some patients.

In addition to the cardinal symptoms of heartburn and regurgitation, dyspeptic symptoms occur frequently in individuals with GERD [6]– [11], including those who are partial responders to PPI [12]. A better understanding of symptom patterns in partial PPI responders with GERD is needed to understand the genesis of symptoms and to improve medical management in this patient group. The aim of this analysis was to compare the symptom pattern in patients with GERD who were partial responders to PPIs with the symptom pattern of treatment-naïve patients with GERD before and after they received treatment with a PPI.

## Methods

### Study population

The *post hoc* analysis was based on two study populations: one from the patient-reported outcome (PRO) Validation Study (ClinicalTrials.gov number NCT00703534), which evaluated the Reflux Symptom Questionnaire electronic Diary [12], and one from the Diamond study (ClinicalTrials.gov number NCT00291746) [13].

The PRO Validation Study was conducted between May and December 2008, and included patients with a partial symptomatic response to PPI therapy. PPI therapy was individually optimized according to the physician’s judgment, within the approved dose range for any GERD indication. Patients were eligible if they had a history of GERD symptoms for at least 6 months, and had experienced at least mild heartburn and/or regurgitation on 3 or more days in the week before the study despite having received at least 4 weeks of PPI therapy. Patients whose symptoms did not improve at all with PPI therapy (i.e. who were non-responders) were excluded, as were individuals who had been receiving twice-daily dosing of PPI therapy and those taking prokinetic drugs.

The Diamond study was conducted between September 2005 and October 2006. It recruited unselected patients presenting with frequent upper gastrointestinal symptoms in primary care. Patients were included if they had not taken a PPI in the previous 2 months, had upper gastrointestinal symptoms of any severity on 2 or more days per week for at least 4 weeks, and had gastrointestinal symptoms of at least mild severity on 3 or more days in the week before study entry. GERD was diagnosed if at least one of the following three criteria was met: reflux esophagitis on endoscopy (Los Angeles grades A–D); pathological distal esophageal acid exposure (esophageal pH < 4 for > 5.5% of the time over 24 hours); and a positive symptom–acid association probability (> 95%). Patients in the Diamond study received esomeprazole 40 mg once daily for 2 weeks as a treatment trial.

The PRO Validation study and the DIAMOND study were both multicenter studies, and both were approved by a central or local Institutional Review Board (IRB)/Research Ethics Committee within each country.

### Gastrointestinal symptom rating scale

Participants in both studies were asked to complete the Gastrointestinal Symptom Rating Scale (GSRS) at the start of the study. In addition, participants in the Diamond study completed the GSRS after 2 weeks of PPI treatment. The GSRS is a PRO instrument that assesses gastrointestinal symptoms using a 7-grade Likert scale, ranging from 1 (‘no discomfort at all’) to 7 (‘very severe discomfort’) [14]. The GSRS consists of 15 items, clustered into 5 domains: Reflux (heartburn, regurgitation); Abdominal pain (abdominal pain, hunger pains, nausea); Indigestion (rumbling, bloated, burping, passing gas); Diarrhea (diarrhea, loose stools, urgent need for bowel movement); and Constipation (constipation, hard stools, feeling of incomplete bowel movement). The GSRS has been extensively psychometrically validated in patients with reflux disease and a within-group score change of 0.5 in 1 of the 5 domains is considered clinically relevant [15].

### Statistical analyses

Mean item and domain discomfort scores of the GSRS were calculated together with the corresponding 95% confidence intervals (CIs).

## Results

### Patients

This analysis included 580 partial PPI responders on PPI therapy from the PRO Validation Study and 203 PPI treatment-naïve patients with GERD from the Diamond study. Demographic characteristics of the 2 groups of patients are presented in Table 1. The groups were similar in terms of age and body mass index (BMI), but the partial PPI responder group had a larger proportion of women and a longer mean history of GERD symptoms than the treatment-naïve group.

**Table 1:** **Demographic characteristics of partial PPI responders and treatment-naïve patients with GERD**

Characteristic	Partial PPI responders	Treatment-naïve patients
	(n = 580)	(n = 203)
Age, years		
Mean (SD)	48.2 (11.5)	47.7 (13.1)
Range	19–70	18–76
Sex, n (%)		
Female	338 (58.3)	87 (42.9)
Male	242 (41.7)	116 (57.1)
Body mass index, kg/m^2^		
Mean (SD)	28.6 (3.8)	27.4 (4.5)
Range	18.9–40.8	15.6–45.7
History of GERD symptoms, years		
Mean (SD)	8.9 (8.1)	4.7 (7.9)
Range	0.5–53.0	0.0–60.0

SD, standard deviation.

### GSRS scores

**Figure 1:**
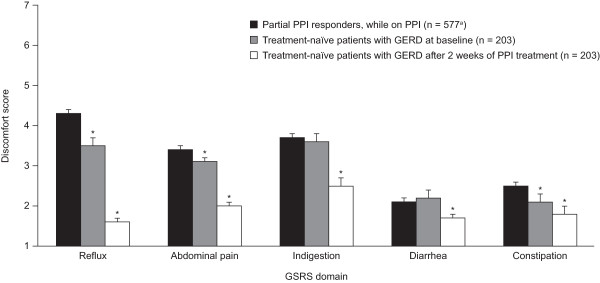
**GSRS discomfort scores in partial PPI responders and in treatment-naïve patients with GERD before and after 2 weeks of PPI therapy.** PPI treatment comprised esomeprazole 40 mg once daily for 2 weeks. The degree of discomfort was rated on a 7-point Likert scale (1, no discomfort at all; 7, very severe discomfort). Mean (95% CI) values are shown. ^a^GSRS data were unavailable for 3 patients in the partial PPI responders group. ^*^Lack of 95% CI overlap vs. partial PPI responders.

Mean (95% CI) GSRS scores in partial PPI responders while on PPI therapy, and in treatment-naïve patients with GERD before and after 2 weeks of PPI therapy, are shown in Table 2 (domain and item scores) and Figure 1 (domain scores only). Partial PPI responders on PPI therapy reported more discomfort in the Reflux, Abdominal pain, and Constipation domains than did treatment-naïve patients before PPI therapy. Symptom scores in partial PPI responders were lower in the Abdominal pain and Constipation domains than in the Reflux and Indigestion domains of the GSRS. The highest mean discomfort scores were reported in the Reflux, Indigestion, and Abdominal pain domains of the GSRS, in partial PPI responders while they were on PPI therapy and in treatment-naïve patients before they received PPI therapy.

**Table 2:** **GSRS discomfort scores in partial PPI responders and in treatment-naïve patients with GERD before and after 2 weeks of PPI therapy**

	Partial PPI responders	Treatment-naïve patients	
GSRS domain/item		Before PPI treatment	After 2 weeks of PPI treatment
	(n = 577 ^a^)	(n = 203)	(n = 203)
Reflux domain	4.3 (4.2–4.4)	3.5 (3.3–3.7)	1.6 (1.5–1.7)
Heartburn	4.4 (4.3–4.5)	3.7 (3.4–3.9)	1.6 (1.5–1.8)
Regurgitation	4.2 (4.1–4.3)	3.3 (3.0–3.5)	1.6 (1.4–1.7)
Abdominal pain domain	3.4 (3.3–3.5)	3.1 (3.0–3.3)	2.0 (1.9–2.2)
Abdominal pain	4.0 (3.9–4.2)	4.1 (3.9–4.3)	2.3 (2.1–2.5)
Hunger pains	3.5 (3.3–3.6)	3.0 (2.8–3.2)	2.1 (1.9–2.3)
Nausea	2.7 (2.6–2.8)	2.4 (2.1–2.6)	1.7 (1.5–1.8)
Indigestion syndrome	3.7 (3.6–3.8)	3.6 (3.4–3.7)	2.5 (2.3–2.6)
Rumbling	3.3 (3.2–3.4)	3.2 (3.0–3.4)	2.3 (2.1–2.4)
Bloated	3.7 (3.5–3.8)	3.8 (3.6–4.0)	2.6 (2.4–2.8)
Burping	3.8 (3.7–3.9)	3.4 (3.2–3.7)	2.3 (2.1–2.4)
Passing gas	3.8 (3.7–4.0)	3.8 (3.5–4.0)	2.7 (2.5–2.9)
Diarrhea syndrome	2.1 (2.0–2.2)	2.2 (2.0–2.4)	1.7 (1.6–1.9)
Diarrhea	1.9 (1.8–2.0)	2.2 (2.0–2.4)	1.8 (1.6–1.9)
Loose stools	2.1 (1.9–2.2)	2.2 (2.0–2.4)	1.7 (1.6–1.8)
Urgent need for bowel movement	2.4 (2.3–2.5)	2.3 (2.1–2.5)	1.8 (1.6–1.9)
Constipation syndrome	2.5 (2.4–2.6)	2.1 (1.9–2.2)	1.8 (1.6–1.9)
Constipation	2.4 (2.3–2.6)	1.9 (1.7–2.1)	1.7 (1.5–1.8)
Hard stools	2.2 (2.1–2.4)	1.9 (1.8–2.1)	1.6 (1.5–1.8)
Feeling of incomplete bowel movement	2.7 (2.6–2.9)	2.4 (2.2–2.6)	2.0 (1.8–2.2)

PPI treatment comprised esomeprazole 40 mg once daily for 2 weeks. Mean (95% CI) domain and item discomfort scores of the GSRS are shown. The degree of discomfort was rated on a 7-point Likert scale (1, no discomfort at all; 7, very severe discomfort).

^a^GSRS data were unavailable for 3 patients in the partial PPI responders group.

SD, standard deviation.

All GSRS domain scores improved in treatment-naïve patients following PPI therapy (Table 2, Figure 1). Improvements exceeded the minimal clinically relevant score change of 0.5 in all domains except Constipation. The greatest effect of PPI treatment was observed in the Reflux domain. Post-treatment discomfort scores in treatment-naïve patients were markedly lower than the corresponding domain scores in partial PPI responders on PPI therapy.

## Discussion

In this analysis, patients with GERD with a partial response to PPIs reported symptom patterns while on PPIs that were similar in prevalence to those in PPI-treatment-naïve patients with GERD. Both patient groups reported the most severe symptoms in the Reflux, Indigestion, and Abdominal pain domains. However, partial PPI responders had more intense symptoms of heartburn and regurgitation while on PPI therapy than did treatment-naïve patients. All GSRS domain scores improved in treatment-naïve patients following PPI therapy.

A score change of 0.5 on a 7-point scale is generally considered to be clinically meaningful [16, 17]. For the GSRS, this minimally meaningful difference has been validated for within-group comparisons by Talley et al., who showed that a change of 0.5 in the Reflux domain was clinically relevant [15]. In the current study, when comparing GSRS domain scores in partial PPI responders with those in treatment-naive patients before PPI therapy, a score difference greater than 0.5 was observed only in the Reflux domain, with score differences being smaller in the other 4 domains. The clinical relevance of this observation needs to be interpreted with caution, however, because the validity of a minimally meaningful score difference of 0.5 applies primarily to inter-group changes over time and/or after treatment [15]; a minimally relevant score difference for between-group comparisons has not been established for the GSRS. When comparing GSRS domain scores in treatment-naïve patients before and after PPI therapy in the present study, a clinically relevant score change was demonstrated in all domains except for Constipation.

Identifying patients who might not respond to PPIs is a challenge in clinical practice and there are limited data addressing this question. In a study of patients with GERD who were symptomatic on twice-daily PPI therapy, there was no correlation between symptoms and reflux in 75% of reflux episodes [18]. The majority of symptomatic reflux episodes were weakly acidic (pH 4 to 7) and reflux episodes reaching the proximal esophagus were more likely to be symptomatic [18]. In one study, patients with a partial response to a PPI had a longer disease duration and were more likely to be obese (BMI ≥ 30 kg/m^2^) than those who responded [19]. In another study, pH–impedance patterns did not predict the lack of a response to PPIs, but the presence of IBS and a BMI of 25 kg/m^2^ or less were risk factors [20]. In our study, the duration of GERD was longer in partial responders to a PPI than in treatment-naïve patients with GERD, but the BMI was similar in the 2 groups. The Indigestion, Diarrhea, and Constipation domains of the GSRS are markers of IBS. There were no differences between PPI partial responders and treatment-naïve patients in the Indigestion and Diarrhea domains, but Constipation domain scores were higher in partial PPI responders than in the treatment-naïve group before PPI therapy. An association between constipation and reflux disease has been described in the literature but a pathophysiological basis has not been elucidated. In a large cross-sectional study in China, Du et al. reported that constipation was a risk factor for non-erosive reflux disease [21]. While visceral sensitivity in the esophagus remains a possible explanation for persistent symptoms while receiving PPI therapy, the absence of meaningful differences in Indigestion or Diarrhea domain scores between PPI partial responders and treatment-naïve patients suggests that a generalized disorder of gastrointestinal visceral hypersensitivity is unlikely.

Partial PPI response is an important clinical problem and the proper management of patients with GERD who have only a partial response to PPI therapy is still uncertain. Results from a systematic literature review indicate that in partial PPI responders more than 80% of reflux-related symptoms are associated with weakly acidic or alkaline reflux therapy [4]. The likely mechanism by which weakly acidic/alkaline reflux generates symptoms is mechanical stimulation, potentially occurring in combination with esophageal hypersensitivity. Treatment with reflux inhibitors has been explored as a possible alternative to PPI therapy when remaining symptoms are clearly reflux related [22, 23]. Studies of drugs designed to decrease transient lower esophageal sphincter relaxations have, however, shown disappointing results so far [24]– [26]. A few insights have emerged from these trials, such as that mild reflux symptoms in patients on PPI therapy do not appear to respond well to reflux inhibitors [26], whereas moderate or severe symptoms might be more responsive [25, 26].

Our analysis included well-characterized patient populations and the same psychometrically validated questionnaire was used in both comparator groups. One possible limitation of the analysis is that the criteria used to define GERD differed in the two studies, thus potentially introducing a selection bias. In the PRO Validation Study, GERD was defined based on clinical diagnosis and the presence of frequent reflux symptoms. The study may thus also have included patients with visceral hypersensitivity, functional heartburn or dyspepsia. In the Diamond study, GERD was defined based on objective investigative findings (reflux esophagitis on endoscopy, pathological esophageal acid exposure and/or a positive symptom–acid association probability), and patients with functional causes for their symptoms were thus likely to have been excluded. Reflux symptoms were nevertheless prevalent in the Diamond study (about 95% of patients with investigation-based GERD reported symptoms of heartburn and/or regurgitation), with similar patterns to those seen in the partial PPI responders. Regardless, both methods are used to diagnose GERD in clinical practice, making the symptom profiles relevant. Furthermore, despite the methodological limitations of analyzing data from different studies, the patient materials that we have access to are unique and are, to our knowledge, the best currently available for comparing symptom patterns in partial responders and treatment-naïve patients with GERD.

## Conclusion

Gastrointestinal symptom patterns in partial PPI responders were similar to those in treatment-naïve patients with GERD, but partial PPI responders experienced more severe reflux symptoms (heartburn and regurgitation) on PPI therapy than did patients with GERD not treated with PPIs. Symptoms of indigestion and diarrhea, which are markers of IBS, were similar in partial PPI responders and untreated patients with GERD, but partial PPI responders reported more severe constipation than the treatment-naïve group. The main symptom burden of patients with GERD who are partial responders to a PPI continues to be heartburn and regurgitation. The relevance to clinical practice of our results is, first, that partial PPI responders have a similar symptom profile as untreated patients with GERD and thus cannot be identified at baseline. And second, that the symptom burden, even with treatment, is as high in partial PPI responders as in untreated patients with GERD.

## Abbreviations

BMI: Body mass index
CI: Confidence interval
GERD: Gastroesophageal reflux disease
GSRS: Gastrointestinal Symptom Rating Scale
IBS: Irritable bowel syndrome
PPI: Proton pump inhibitor
PRO: Patient-reported outcome.

## References

[1] El-Serag H, Becher A, Jones R (2010). Systematic review: persistent reflux symptoms on proton pump inhibitor therapy in primary care and community studies. Aliment Pharmacol Ther.

[2] Toghanian S, Johnson DA, Stalhammar NO, Zerbib F (2011). Burden of gastro-oesophageal reflux disease in patients with persistent and intense symptoms despite proton pump inhibitor therapy: a post hoc analysis of the 2007 national health and wellness survey. Clin Drug Investig.

[3] Fass R, Shapiro M, Dekel R, Sewell J (2005). Systematic review: proton-pump inhibitor failure in gastro-oesophageal reflux disease – where next?. Aliment Pharmacol Ther.

[4] Boeckxstaens GE, Smout A (2010). Systematic review: role of acid, weakly acidic and weakly alkaline reflux in gastroesophageal reflux disease. Aliment Pharmacol Ther.

[5] Neumann H, Monkemuller K, Kandulski A, Malfertheiner P (2008). Dyspepsia and IBS symptoms in patients with NERD, ERD and Barrett’s esophagus. Dig Dis.

[6] Gerson LB, Kahrilas PJ, Fass R (2011). Insights into gastroesophageal reflux disease-associated dyspeptic symptoms. Clin Gastroenterol Hepatol.

[7] Vakil N, Veldhuyzen van Zanten S, Kahrilas P, Dent J, Jones R (2006). The Montreal definition and classification of gastro-esophageal reflux disease (GERD) – a global evidence-based consensus. Am J Gastroenterol.

[8] Vakil N, Halling K, Ohlsson L, Wernersson B (2013). Symptom overlap between postprandial distress and epigastric pain syndromes of the Rome III dyspepsia classification. Am J Gastroenterol.

[9] Quigley EM, Lacy BE (2013). Overlap of functional dyspepsia and GERD–diagnostic and treatment implications. Nat Rev Gastroenterol Hepatol.

[10] de Bortoli N, Martinucci I, Bellini M, Savarino E, Savarino V, Blandizzi C, Marchi S (2013). Overlap of functional heartburn and gastroesophageal reflux disease with irritable bowel syndrome. World J Gastroenterol.

[11] Savarino E, Pohl D, Zentilin P, Dulbecco P, Sammito G, Sconfienza L, Vigneri S, Camerini G, Tutuian R, Savarino V (2009). Functional heartburn has more in common with functional dyspepsia than with non-erosive reflux disease. Gut.

[12] Vakil N, Björck K, Denison H, Halling K, Karlsson M, Paty J, Silberg D, Rydén A (2012). Validation of the Reflux Symptom Questionnaire electronic Diary in partial responders to proton pump inhibitor therapy. Clin Trans Gastroenterol.

[13] Dent J, Vakil N, Jones R, Bytzer P, Schoning U, Halling K, Junghard O, Lind T (2010). Accuracy of the diagnosis of GORD by questionnaire, physicians and a trial of proton pump inhibitor treatment: the Diamond Study. Gut.

[14] Dimenäs E, Glise H, Hallerbäck B, Hernqvist H, Svedlund J, Wiklund I (1995). Well-being and gastrointestinal symptoms among patients referred to endoscopy owing to suspected duodenal ulcer. Scand J Gastroenterol.

[15] Talley NJ, Fullerton S, Junghard O, Wiklund I (2001). Quality of life in patients with endoscopy-negative heartburn: reliability and sensitivity of disease-specific instruments. Am J Gastroenterol.

[16] Juniper EF, Guyatt GH, Willan A, Griffith LE (1994). Determining a minimal important change in a disease-specific quality of life questionnaire. J Clin Epidemiol.

[17] Guyatt GH, Juniper EF, Walter SD, Griffith LE, Goldstein RS (1998). Interpreting treatment effects in randomised trials. BMJ.

[18] Zerbib F, Duriez A, Roman S, Capdepont M, Mion F (2008). Determinants of gastro-oesophageal reflux perception in patients with persistent symptoms despite proton pump inhibitors. Gut.

[19] Dickman R, Boaz M, Aizic S, Beniashvili Z, Fass R, Niv Y (2011). Comparison of clinical characteristics of patients with gastroesophageal reflux disease who failed proton pump inhibitor therapy versus those who fully responded. J Neurogastroenterol Motil.

[20] Zerbib F, Belhocine K, Simon M, Capdepont M, Mion F, Bruley des Varannes S, Galmiche JP (2012). Clinical, but not oesophageal pH-impedance, profiles predict response to proton pump inhibitors in gastro-oesophageal reflux disease. Gut.

[21] Du J, Liu J, Zhang H, Yu CH, Li YM (2007). Risk factors for gastroesophageal reflux disease, reflux esophagitis and non-erosive reflux disease among Chinese patients undergoing upper gastrointestinal endoscopic examination. World J Gastroenterol.

[22] Fass R, Sifrim D (2009). Management of heartburn not responding to proton pump inhibitors. Gut.

[23] Sifrim D, Zerbib F (2012). Diagnosis and management of patients with reflux symptoms refractory to proton pump inhibitors. Gut.

[24] Vakil NB, Huff FJ, Bian A, Jones DS, Stamler D (2011). Arbaclofen placarbil in GERD: a randomized, double-blind, placebo-controlled study. Am J Gastroenterol.

[25] Shaheen NJ, Denison H, Bjorck K, Karlsson M, Silberg DG (2013). Efficacy and safety of lesogaberan in gastro-oesophageal reflux disease: a randomised controlled trial. Gut.

[26] Vakil NB, Huff FJ, Cundy KC (2013). Randomised clinical trial: arbaclofen placarbil in gastro-oesophageal reflux disease–insights into study design for transient lower sphincter relaxation inhibitors. Aliment Pharmacol Ther.

[CR27] The pre-publication history for this paper can be accessed here: http://www.biomedcentral.com/1471-230X/14/177/prepub

